# Supplementary figures and images for: Systematically identification of survival-associated eQTLs in a Japanese kidney cancer cohort
Source: PLoS Genet. 2025 Jul 7;21(7):e1011770. doi: 10.1371/journal.pgen.1011770 (PMC12233309; doi:10.1371/journal.pgen.1011770)

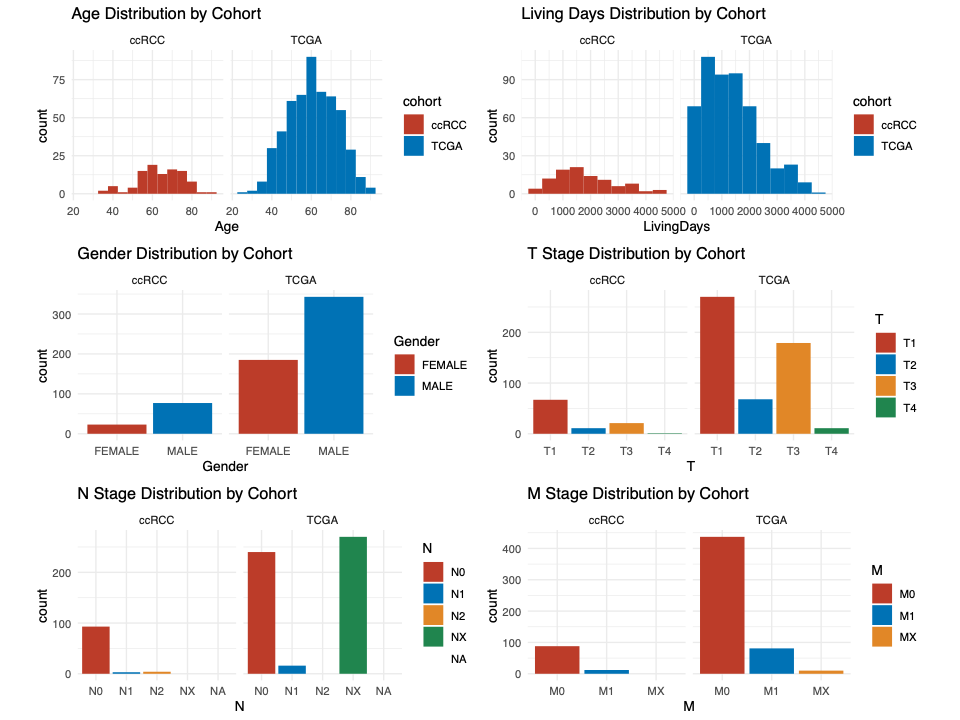

Supplement: S1 Fig — This includes age, gender, living days, stage at diagnosis (T), lymph node spread (N), and metastases stage (M). (TIF) [file pgen.1011770.s001.tif]

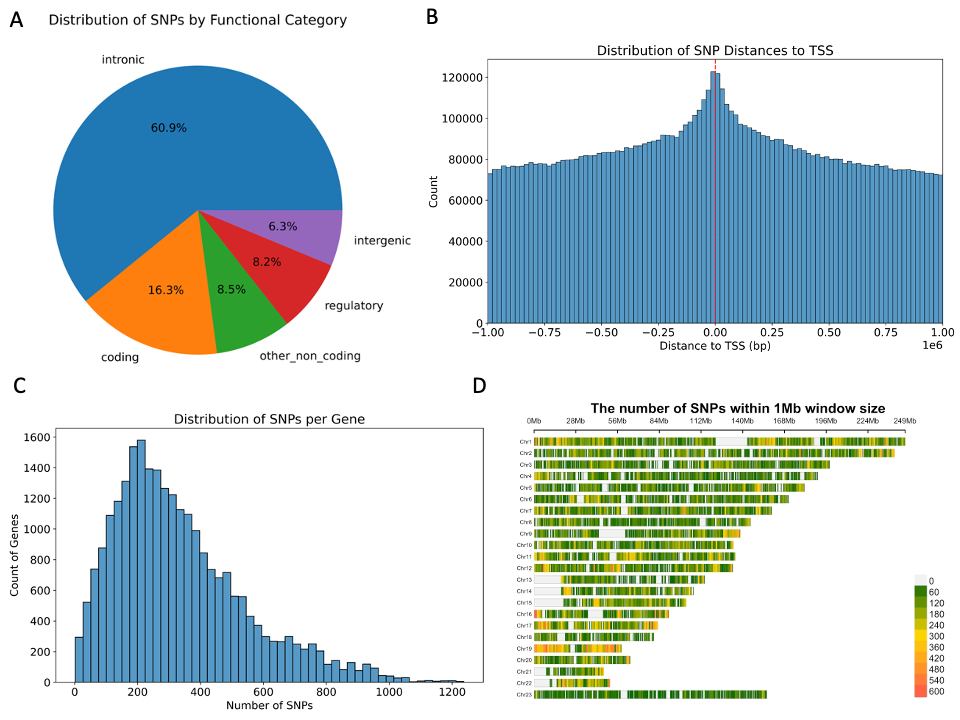

Supplement: S2 Fig — (A) Proportions of variants in intronic (60.9%), coding (16.3%), and regulatory (8.2%) regions. The “coding” category in the plot includes variants annotated as missense, stop gained/lost, start lost, frameshift, splice site, inframe insertion/deletion, protein-altering, transcript ablation/amplification, and feature elongation/truncation. The “regulatory” category includes variants in 5′ and 3′ untranslated regions (UTRs), promoter regions, enhancers, transcription factor binding sites, as well as upstream and downstream gene variants. The “intronic” category includes intron variants, while “intergenic” refers to variants annotated as intergenic. Variants not fitting these classifications were grouped as “other non-coding”. (B) Distribution of variant distances from transcription start sites (TSSs). (C) Count distribution of variants per gene. (D) Chromosome-level density plot showing genome-wide distribution of tested variants. (TIF) [file pgen.1011770.s002.tif]

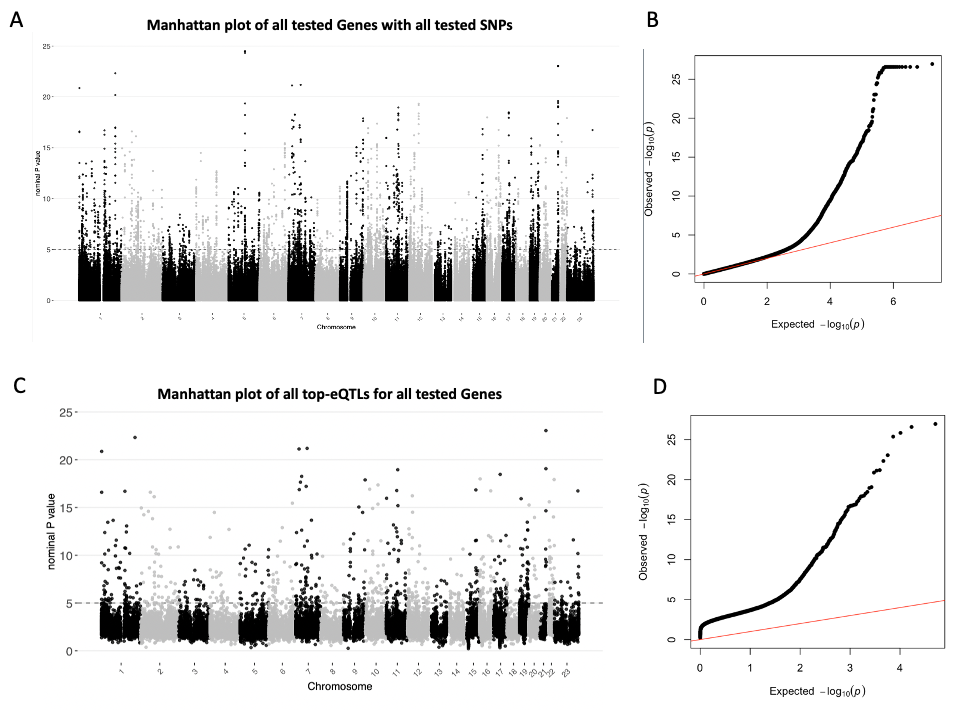

Supplement: S3 Fig — This figure shows the Manhattan plot and Q-Q plot of cis-eQTL analyses in the JP cohort. A total of 25,508 genes and 8,495,717 gene-SNP/Indel pairs were analyzed. A total of 180 genes without variant information were excluded from the analysis. (TIF) [file pgen.1011770.s003.tif]

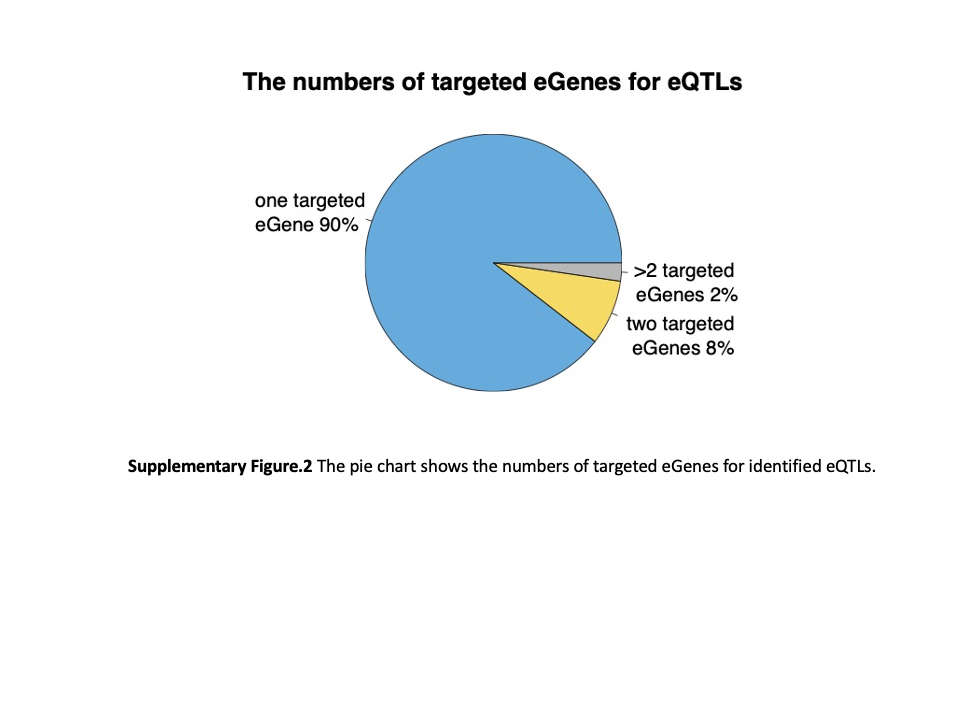

Supplement: S4 Fig — Among 4,558 unique eQTLs, 90% target one eGene and 10% target multiple eGenes. (TIF) [file pgen.1011770.s004.tif]

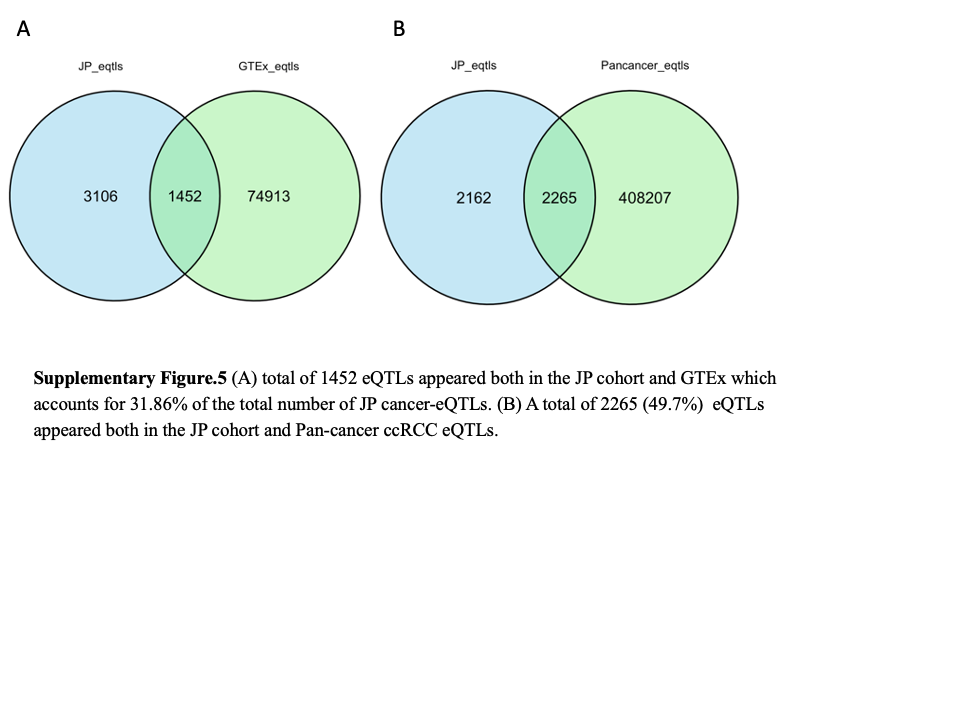

Supplement: S5 Fig — (A) Venn diagram showing 1,452 (31.86%) eQTLs shared with GTEx. (B) Comparison showing 2,265 (49.7%) JP eQTLs overlap with Pan-cancer ccRCC eQTLs. (TIF) [file pgen.1011770.s005.tif]

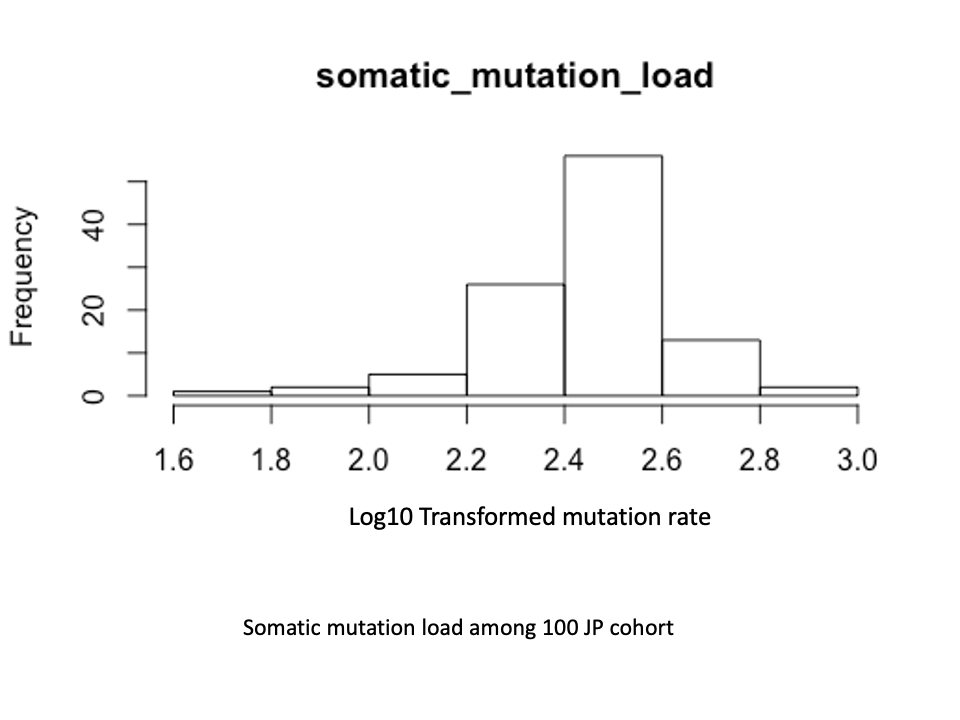

Supplement: S6 Fig — Over 40% of samples show a log10-transformed mutation load between 2.4 and 2.6. (TIF) [file pgen.1011770.s006.tif]

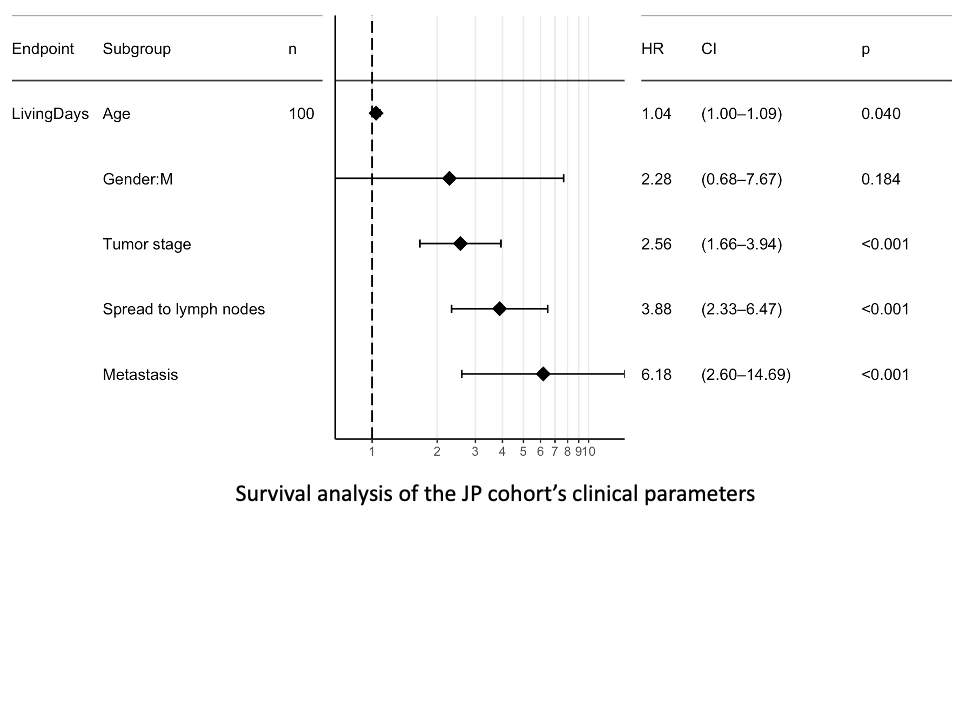

Supplement: S7 Fig — This Figure shows the analysis results for age, gender, tumor stage (T), lymph node spread (N), and metastasis (M). Age and gender were not significantly associated with overall survival (OS), while tumor stage, lymph node spread, and metastasis showed significant associations with OS (P < 0.001). (TIF) [file pgen.1011770.s007.tif]

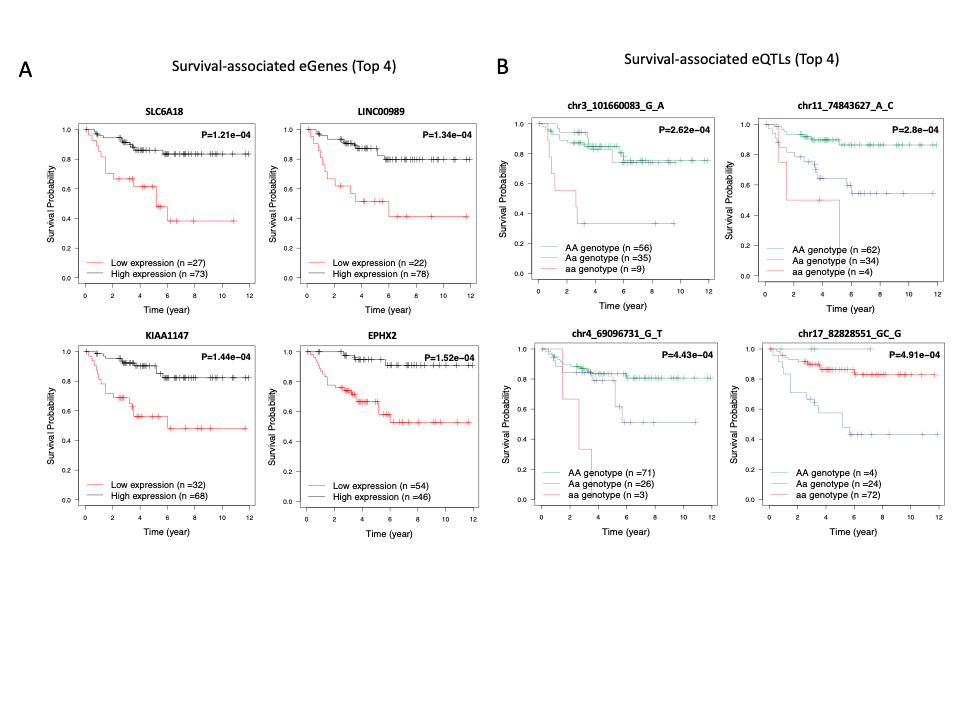

Supplement: S8 Fig — These plots visualize the prognostic effects for the top 4 eGenes and eQTLs ranked by log-rank P-value. Each plot highlights the significant association between gene expression or variant status and overall survival in the JP cohort. (TIF) [file pgen.1011770.s008.tif]

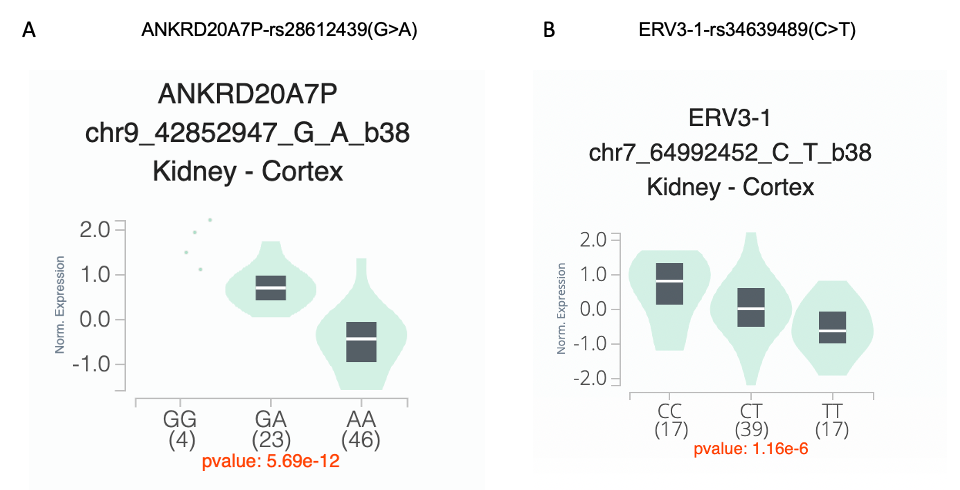

Supplement: S9 Fig — These two eSNPs show the same allelic effects towards gene expression patterns investigated from the JP cohort. (TIF) [file pgen.1011770.s009.tif]

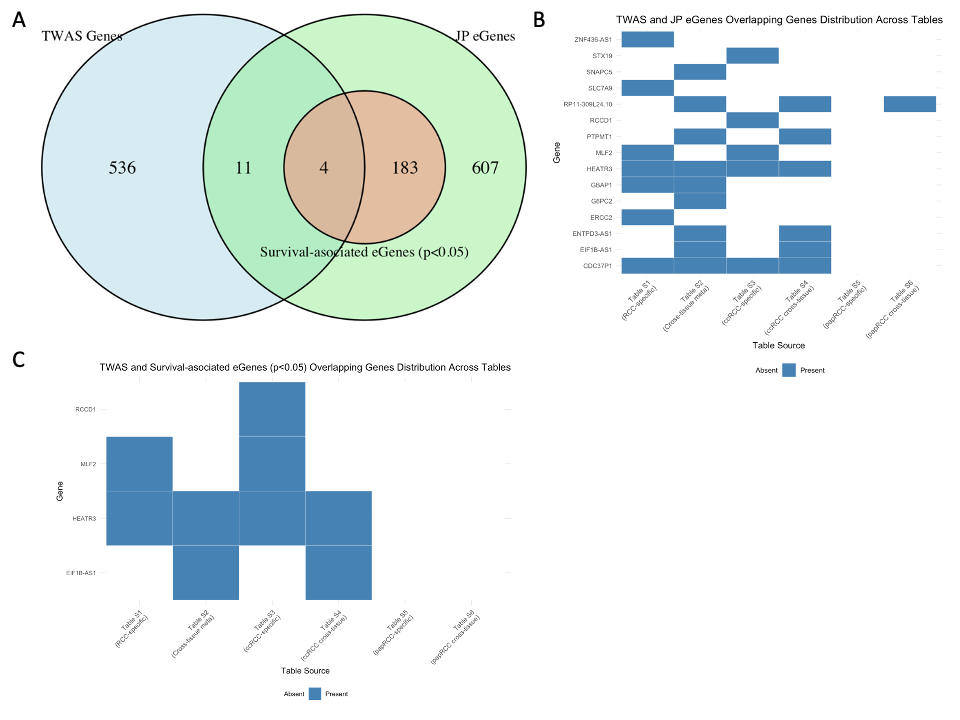

Supplement: S10 Fig — (A) Fifteen overlapping genes were identified between JP eGenes and TWAS genes, including 11 general overlaps and 4 prognostic overlaps. (B) Distribution of these 15 overlapping genes across analysis tables. (C) Four genes overlap between TWAS genes and JP prognostic eGenes,. (TIF) [file pgen.1011770.s010.tif]
