# Supplementary material for: Systematically identification of survival-associated eQTLs in a Japanese kidney cancer cohort
Source: PLoS Genet. 2025 Jul 7;21(7):e1011770. doi: 10.1371/journal.pgen.1011770 (PMC12233309; doi:10.1371/journal.pgen.1011770)
Supplement: S1 Table — This table shows the number of variants remaining at each QC step. A total of 284,774 variants passed QC filters, including 244,803 SNPs, 17,346 insertions, and 22,625 deletions. Of these, 283,376 variants (~99.5%) within ±1 Mb windows around gene TSSs were used for eQTL analysis. (DOCX) [file pgen.1011770.s011.docx]

| **Filtering / processing criterion** | **Total sites** | **Percentage** |
| --- | --- | --- |
| Initial variants | 7088610 | 100.00% |
| VQSR and ExcessHet not PASS | 6813720 | 96.12% |
| QD <2.0 or QUAL <30 | 6804941 | 96.00% |
| InbreedingCoeff < -0.3 | 6802902 | 95.97% |
| Hail splitting Multi-allelic sites | 6927794 | / |
| Monomorphic sites with AC =0 | 6356484 | 89.67% |
| Missingness > 25% | 459062 | 6.48% |
| MAF <=1% | 284774 | 4.02% |

**Supplementary Table 1**. Variants QC filtering from Haplotypecaller
