# Supplementary material for: Systematically identification of survival-associated eQTLs in a Japanese kidney cancer cohort
Source: PLoS Genet. 2025 Jul 7;21(7):e1011770. doi: 10.1371/journal.pgen.1011770 (PMC12233309; doi:10.1371/journal.pgen.1011770)
Supplement: Attachment — Submitted filename: Response to Reviewers_PGENETICS-D-25-00018.docx [file pgen.1011770.s021.docx]

Dear editors,

We would like to thank you and the reviewers for the thorough evaluation of our manuscript (Manuscript Number: PGENETICS-D-25-00018) titled "Systematically identification of survival-associated eQTLs in a Japanese kidney cancer cohort".

1. We provided point-by-point responses to the reviewers' comments and updated the corresponding main Figures, supplementary Figures, tables, references, abstract, and the main text in the Methods, Results, and Discussion sections. The main changes includes:

(1) We clarified the major concerns regarding our tested variants in the scanning windows, the variants included in WES-based eQTL analysis, and provided detailed explanations of identified regulatory signals from intronic regions.
(2) We addressed the multiple testing results, showed that the prognostic eGenes satisfied multiple correction (Table 2), and clarified our methodologies of exploratory analysis based on our hypothesis and cross-cohort validation.
(3) We performed extra sensitivity analysis suggested by reviewer #3 to ensure we weed out false positive results due to limited sample size. We also confirmed that our major results were not affected after performing this analysis.
(4) We performed extra analyses and figures suggested by reviewer #3, such as somatic figures, minor allele frequencies with effect size plot, a lenient search for TCGA replicates based on the same direction of prognostic effects, comparison with other studies, etc. We also discussed the points raised by reviewer #3 regarding population-specific eQTL signals in the JP cohort.
(5) We added the limitations section to acknowledge the limitations that reviewers #1 and #2 mentioned.

2. Revised the manuscript to comply with the journal's submission requirements.

3. Prepared and included the full summary statistics of the eQTL analysis, in accordance with the editors’ advice.

We believe these revisions have greatly improved the manuscript, making it more focused and scientifically robust. We hope that the revised version meets the expectations of both the editors and the reviewers. We look forward to hearing from you and are prepared to make any further revisions as needed.

Thank you once again for the opportunity to revise and improve our work.

Sincerely,

Adil Mardinoglu	Cheng Zhang

Professor of Systems Biology	Lecturer, Researcher

[adilm@scilifelab.se](mailto:adilm@scilifelab.se)	[cheng.zhang@scilifelab.se](mailto:cheng.zhang@scilifelab.se)

adil.mardinoglu@kcl.ac.uk

SciLifeLab, Science for Life Laboratory,	SciLifeLab

KTH-Royal Institute of Technology,	KTH

Stockholm, Sweden	Stockholm, Sweden

Centre for Host-Microbiome Interactions	The Roger Williams Institute of Liver	Studies

Faculty of Dentistry, Oral & Craniofacial Sciences	Faculty of Life Sciences & Medicine

King's College London, UK	King's College London, UK

http://sysmedicine.com

**Journal Requirements:**

1) We confirmed the author contributions are listed correctly.

2) We have added author summary in our revised manuscript.

3) We have prepared main Figs as tif format generated from  Preflight Analysis and Conversion Engine (PACE).

4) We have prepared a list of Suplementary Information, placed after refereces and main tables.

5) We have revised the Funding section to include the role of funders took in the study.

**Point-to-point response to reviewers’ comments:**

We would like to thank all of the reviewers for their valuable comments and constructive suggestions. We have addressed reviewers' comments below with our point-to-point response in blue font and made changes in manuscripts and corresponding Figs/tables. All changes in the main text are tracked.

**Reviewers' comments:**

**Reviewer #1**: In this study, authors applied the previously published data of whole-exome sequencing and RNA sequencing data from 100 Japanese
ccRCC patients to identify eQTLs. The prognostic effect of the expressed genes was validated using public TCGA data of 287 patients.

The advantage of this study is a series of bioinformatic methods used to do this.

We sincerely appreciate your time spent reading our work and your valuable insights regarding the bioinformatic approaches we applied for intergrative analysis.

The disadvantage included (1) prognosis was affected by many therapies and demographic information for the patients. These factors should be all included in Cox proportional model; (2) whole-exome sequencing only includes a percentage of functional SNPs in the promoter region and/or enhancer regions. The data od eQTLs are not intact. (3) functional experiments should be helpful to enrich the evidences.

Thank you for highlighting these important limitations in our study. In response to your valuable suggestions:

(1) prognosis was affected by many therapies and demographic information for the patients. These factors should be all included in Cox proportional model:

We fully agree that the prognosis of cancers is affected by numerous factors in real-world, including therapies and demographic information. However, in a majority of clinical cancer datasets, such information is often incomplete, and therapy details are inconsistently recorded across patients and cohorts. In our cases, most therapy variables and several demographic fields were >80 % missing in both cohorts, making reliable adjustment impossible. We therefore fitted the Cox models with the consistently recorded covariates (age, sex, and TNM stage) and interpreted the resulting hazard ratios as treatment- and demography-independent prognostic effects. This limitation is now stated explicitly in the limitations in discussion: “*Firstly, survival models in this study did not include the therapies that patients underwent due to limitations in the metadata.*”

We also sincerely appreciate the opportunity to clarify the methodological rationale for our survival analyses in this study:

We explored survival associations using both univariate and multivariate Cox models. Adjusted models included available clinical covariates (age, sex, and tumor stage), aiming to control for confounding and to identify direct prognostic effects with potential causal relevance. In contrast, univariate models enabled broader biomarker discovery, which may reflect indirect mechanisms, as commonly applied in previous studies (2)(3)(4)(5). These approaches might be helpful for not missing out on any valuable prognostic-associated eQTLs/eGenes. Lastly, we also performed cross-cohort validation further prioritized robust signals across patient populations.

**(**2) whole-exome sequencing only includes a percentage of functional SNPs in the promoter region and/or enhancer regions. The data od eQTLs are not intact.

Firstly, we fully agree and appreciate your comment on this point, and we acknowledge this limitation in our study. We have stated this point as one of the limitations in the discussion section*:* “*Secondly*, *WES data covers only a limited subset of functional regulatory regions at non-coding sites. Some significant regulatory variants might be missing in the results.*”

Secondly, the variant calling pipeline we used in this study tried to utilized both on-target and off-target sequencing regions to include more potential regulatory variants for downstream analysis. We believe that this WES dataset remains highly valuable, especially given the challenges of collecting kidney cancer tissue samples from patients.

We implemented this approach by solid literature and study background. Prior studies (6)(7)(8) show that WES platforms, including the Agilent SureSelect v4 for the JP cohrot capture substantial off-target regions (often>50% of sequenced bases), including promoters, UTRs, and exon-flanking introns. Especially, Guo et al. (6) noted that "a significant amount of high quality genotypes outside target regions can be obtained." In our study, we also included off-target regions with sufficient coverage and variant quality to perform downstream eQTL analysis. Overall, we believe our work provides valuable insights into regulatory mechanisms relevant to ccRCC by utilizing WES data. We have revised our manuscript to explain this methods on section 2.3: “*To utilize the WES data to explore regulatory effects on non-coding regions, the variant calls were kept if passing quality control, regardless in WES-exon target regions or in off-target regions.”*

Further discussions about WES technologies and the details of captured variants included in this study can be found in our responses to reviewers #2 and #3.

(3) functional experiments should be helpful to enrich the evidences.

We acknowledge that this is a purely bioinformatics study that was not able to perform functional validation experiments. Our intention is to provide computational evidence that can guide and prioritize future experimental work by wet lab groups. By identifying promising genetic factors associated with survival outcomes, we hope to contribute to the broader cancer research ecosystem by highlighting potential targets for functional studies. We have clarified this limitation in the revised manuscript: “*The regulatory signals identified in this study have not yet been subjected to functional experiments, rather serve as a reference database for future studies.*”

**Reviewer #2**: In this manuscript, Song and colleagues performed an eQTL analysis of kidney cancer tissues of a Japanese population and assessed the association of the eQTL genes/variants with cancer outcome in the same cohort. This will be a good resource for kidney cancer genetics research by adding a dataset representing East Asian populations. I have the following comments for the authors:
Major:
1. One of the main limitations of the dataset is that the genotypes are based on exome sequencing data with limited coverage beyond coding regions. The authors mentioned that variants in +/-1Mb of the TSS were used for the regression, but the coverage beyond immediate upstream of TSS or far downstream beyond transcription end site will be sparse with only coding regions of neighboring genes are covered. The authors also mentioned that “the missing genotypes were imputed by FastQTL” (line 223), but it is not clear what reference dataset and what methods were used. Moreover, it is not clear to what extent within +/-1Mb window of the TSS the variants were imputed and used for the analysis, and if imputation quality score cutoff was applied. Did the authors mean they imputed non-coding variants that were not originally covered in the WES panel?

We thank the reviewer for these detailed comments. We would like to clarify that we did not perform large-scale genotype imputation using external reference panels. In our study, we used only the variants directly genotyped from WES data following GATK best practice, which covers coding regions and substantial proximal non-coding regions from the high-quality variant calls from off-target capture (as supported by previous studies (6)(7)(8)). The imputation of FastQTL was used only to fill in missing genotype calls among the variants from the given input genotype files. These missing genotype calls were imputed to the mean by FastQTL (https://github.com/francois-a/fastqtl/issues/23) without the use of an external reference panel. Although FastQTL’s imputation does not generate quality scores in the same way as reference-based tools, we scrutinized the imputed genotypes to include only those with robust performance, as evidenced by consistency in genotype calls across samples.

We now have revised the sentence referring to "missing genotypes imputed by FastQTL" to “*The missing genotypes were imputed to mean dosage across samples by FastQTL within the regression model. No external genotype imputation was performed.*”

Please provide the detailed criteria and quality control in dealing with the missing genotypes within the +/-1Mb scanning window.

Prior to running FastQTL, we applied stringent quality filters, retaining only variants with less than 25% missingness among 100 samples, and we ensured that each genotype call had passed our quality controls (QC) as we stated more clearly in our section 2.3 Data pre-processing . We also showed the number of remaining variants of the JP cohort in each step of quality control in our original **Supplementary Table 1**.

| **Filtering / processing criterion** | **Total sites** | **Percentage** |
| --- | --- | --- |
| Initial variants | 7088610 | 100.00% |
| VQSR and ExcessHet not PASS | 6813720 | 96.12% |
| QD <2.0 or QUAL <30 | 6804941 | 96.00% |
| InbreedingCoeff < -0.3 | 6802902 | 95.97% |
| Hail splitting Multi-allelic sites | 6927794 | / |
| Monomorphic sites with AC =0 | 6356484 | 89.67% |
| Missingness > 25% | 459062 | 6.48% |
| MAF <=1% | 284774 | 4.02% |

**Supplementary Table 1**. Variants QC filtering from Haplotypecaller

After the variant quality control, a total of 284,774 variants (including 244,803 SNPs, 17,346 insertions, and 22,625 deletions) were retained from the WES data. Of these, 283,376 variants (~99.5%) fell within the ±1 Mb windows around gene TSSs and were included in the eQTL analysis (eQTL-ccRCC-final.allpairs.txt.gz). This high proportion is expected given the design of whole-exome sequencing (WES), which enriched coding exons and substantial flanking regions, including UTRs, proximal introns, and nearby regulatory sequences. We have revised our results section 3.1 to clearly stated the variants included in the +/-1Mb scanning window: “*A total of 284774 variants were kept after variants QC filtering, including 244803 SNPs, 17346 insertions, and 22625 deletions. Of these, 283,376 variants (~99.5%) fell within the ±1 Mb windows around gene TSSs and were included in the FastQTL analysis*.”

Please also provide data on distribution of the SNPs included in eQTL analysis within this window.

To address your concerns about the composition of variants within the ±1 Mb window, we have now included supplementary data that details the distribution of variants across this region, as revised in the manuscript: “Inside ±1 Mb windows, 60.9% of tested variants falls in intronic region, 16.3% of them in coding region and 8.2% in regulatory region(**Supplementary Fig 2A**). **Supplementary Fig 2B** displays the distribution of variant distances from TSSs. The count distribution of tested variants per gene is shown in **Supplementary Fig 2C**. A chromosome-level density plot depicting the overall genomic distribution of tested variants is also provided as **Supplementary Fig 2D**”.

This additional information should provide a clear picture of the variant landscape used in our eQTL analysis. We trust that these clarifications address the reviewer’s concerns regarding both the imputation methodology and the handling of missing genotype data in our study.

2. One of the important considerations of eQTL analysis using tumor tissues is the copy number adjustment. In the regression model, the authors did not account for gene expression differences in the tumor transcriptome resulting from copy number changes in the tumor genome. If the somatic copy number information (e.g., gene-level copy number) is not available, please state this as a limitation.

Thank you for this important point about eQTL analysis in tumor tissues. We fully agree with your observation regarding copy number adjustment. We didn't have access to somatic copy number information for either cohort. We have clearly acknowledged this in our discussion section: “*Cancer tissue transcriptomics might be affected by copy number variations (CNVs) that are generated in tumor tissue cells, which we were not able to take into account due to data limitations.”*

We also noted that several published cancer eQTL studies have faced similar constraints and were also unable to account for effects of tumor CNVs on gene expression in their methodologies(9)(10)(11). This represents a common challenge in the field that warrants attention in future studies as more comprehensive genomic data becomes available.

3**.** The authors tested 805 eGenes and 4,558 cis-eQTL variants for their correlation with survival in their cohort but did not mention any multiple testing corrections for the number of genes and variants tested. Please apply proper corrections before declaring significance of prognostic eGenes/eQTLs.

We thank the reviewers for their attention to the issue of multiple testing correction.

Following your suggestion, we now report FDR-corrected results for all 805 eGenes (BH procedure): 7 remain significant in univariate and 3 in multivariate models (**Table 2**). None of the 4 558 eQTLs survives FDR adjustment, which is expected—the variants influence survival only indirectly through gene expression and each effect is correspondingly small. Uncorrected (P < 0.05) eQTL signals are therefore presented only as exploratory leads; we highlight those that show concordant gene-level effects and replicate in TCGA.

We have revised the results section 3.4 accordingly: “*In the context of eGenes, after multiple testing correction by BH procedure, there are 7 solid prognostic eGenes identified in the univariate Cox analysis and 3 prognostic eGenes in adjusted Cox analysis (****Table 2****).*” And also “*In the context of eSNPs, there were no significant prognostic signals on the threshold of FDR<0.05.”*

Table 2. The solid prognostic eGenes from Cox analysis of JP cohort (Unadjusted & Adjusted)

| **gene_name** | **COX_coef** | **P** | **FDR** | **mean_TPM** | **simple_gene_id** | **gene_biotype** | **Models** |
| --- | --- | --- | --- | --- | --- | --- | --- |
| HEATR3 | 0.118081510803151 | 0.000101734787438635 | 0.0117521989575024 | 14.5714431 | ENSG00000155393 | protein_coding | Unadjusted Cox  (Prognosis ~ eGenes) |
| TUBA1C | 0.0136919860126823 | 0.000102193034413064 | 0.0117521989575024 | 48.716941 | ENSG00000167553 | protein_coding |  |
| RP11-218F10.3 | 0.516706512257168 | 0.000133331833116278 | 0.0134165157073255 | 1.09183491 | ENSG00000273449 | lncRNA |  |
| CPNE1 | 0.0124393189847147 | 0.000295297023621351 | 0.0264126782239097 | 89.079656 | ENSG00000214078 | protein_coding |  |
| MMP16 | 0.145675293952334 | 0.000333449662722653 | 0.0268426978491736 | 1.18333664 | ENSG00000156103 | protein_coding |  |
| RAET1E | -3.47214258299286 | 0.000451638384273497 | 0.0330517181218332 | 0.50258754 | ENSG00000164520 | protein_coding |  |
| GEN1 | 0.306880495210678 | 0.000583502480514238 | 0.0391432914011635 | 3.09747835 | ENSG00000178295 | protein_coding |  |
| CENPU | 0.0873760607542322 | 0.000300868001600201 | 0.0453022028183162 | 8.39568621 | ENSG00000151725 | protein_coding | Adjusted Cox (Prognosis ~ eGenes +sex+age+TNM ) |
| STMP1 | 0.0843195495340997 | 0.000186453679484458 | 0.0375238029962471 | 27.600688 | ENSG00000243317 | protein_coding |  |
| TUBA1C | 0.0154766850518572 | 0.000337656170074406 | 0.0453022028183162 | 48.716941 | ENSG00000167553 | protein_coding |  |

However, we would like to emphasize the following points to explain our methodological choices on the further explorary analysis of eSNPs:

We would like to clarify the rationale behind our exploratory analysis of eSNPs:
(1) Selection of regulatory variants: We focused on eQTL variants that passed FDR correction (q < 0.05) in gene expression association analysis. These 4,558 cis-eQTLs showed significant regulatory signals on gene expression and were selected as biologically relevant candidates, rather than random SNPs.
(2) Biological consistency: Beyond statistical significance, we explored eQTL–eGene pairs showing consistent prognostic directions. Specifically, we hypothesized that eQTLs and their target eGenes would tend to show concordant survival effects — positive slopes in favorable genes corresponding to protective eQTLs, and vice versa. This approach identified 223 eQTLs forming 329 pairs of consistent associations across 54 eGenes.
(3) Cross-cohort consistency exploration: To further explore robustness, we examined whether the same alleles showed consistent prognostic associations and effect directions across two independent cohorts (Japan and TCGA). While not all signals replicated, this directional consistency provided an additional layer to prioritize potential candidate eGene-eQTL pairs.
(4) Finding eQTL–eGene pairs with survival relevance is challenging due to the complexity of gene regulation and tumor prognosis. Our multi-step strategy may help reveal additional potential regulatory signals beyond those detectable by strict statistical thresholds alone.

In the revised manuscript, we have moved all downstream analysis that based on p-value instead of q-value as an exploratory analysis for potential prognostic signals, especially those consistent on eGenes and eSNPs pairwise-level and consistent in two cohort: “*We further performed a novel approach of exploratory analysis of eGene-eSNPs pairs with their potential prognostic effects, especially on the hypothesis that important eGene-eSNPs pairs would demonstrate consistent prognosis patterns at both gene and SNP levels. Furthurmore, we aimed to find those consistent pairs across two independent cohorts. The hypothesis and results were summarized* ***in Fig 5A-B****. In this approach, we kept signals with pvalue <0.05 as explorable candidates.*”

Lastly, we revised our abstract accordingly*:” Survival analysis revealed a total of 10 eGenes significantly associated with overall survival (FDR<0.05). Further exploratory analysis were performed using 158 eGenes and 711 eQTLs (P value <0.05) as potential prognostic signals.”*

For your interest, we also improved our exploratory analysis based on suggestions from Reviewer #3.

4. Since this dataset could serve as a resource for the community, please share the full eQTL summary stats.

Yes, the full eQTL summary stats have been shared as a zenedo repository with DOI No. [10.5281/zenodo.15333222](https://doi.org/10.5281/zenodo.15333222), for review process. They will be open to public with the publishment. We have added the statement in “Data Availability” section. A link is created for viewing the whole repo: https://zenodo.org/records/15333223?token=eyJhbGciOiJIUzUxMiIsImlhdCI6MTc0NjYxODUwNCwiZXhwIjoxNzUyMTA1NTk5fQ.eyJpZCI6Ijg5Y2Y3MmJhLTBmZGQtNDdhZS1hOTE0LTg4ZDllZGFjYWRjNSIsImRhdGEiOnt9LCJyYW5kb20iOiIyMGRjMTJjY2U2YTg0YjlmYjE4YTBiYTgwY2IzZDliZiJ9.M1yqlQAwFb1sWEohDeyJAObkBmy5s0ZmwzIqTSCE61Scx_1keTdcY8gJ2NlcMo9-IV2XVlhQYPtUPvc0TCjrSA

Minor:
1. Line 222: Permutation for eQTL adjusted p-value was mentioned as 100 to 1000 times but shown as 1000 to 10000 in Fig 1

Thank you for pointing out this typo error in the text description. We have revised the texts to the correct permutation which is 1000 to 10000 as the Fig 1 showed: “*An adaptive permutation pass between 1000 to 10000 times was used to calculate the adjusted p-value for each gene’s top associated variant*”.

2. Line247: please specify that gene expression levels (the same normalized expression levels used in the eQTL study?) was used for the association between eGenes and overall survival to clarify the analysis for the readers.

Thank you for your suggestion on this point. We have accordingly clarified and revised the manuscript: *“Univariate survival analysis by the Cox proportional hazards regression model (Cox) was performed in the JP cohorts, using the same TPM-normalized gene expression data employed in the eQTL study, to evaluate the association between previously identified eGenes (n = 805) and corresponding eQTLs (n = 4558) with overall survival (OS).”*

3. Line 258: please clarify whether both the HR and eQTL slope are relative to the minor allele. FastQTL pipeline typically generates slope relative to alternative allele, which might not be the same as the minor allele in this population. Aligning eQTL and survival analysis to the same allele is important given the authors later filter results based on consistency of the direction between eQTL and eGenes.

Thank you for this improtant point. We have confirmed that both the HR (in survival analysis) and the eQTL slope are aligned to the same allele, ensuring proper directional comparison. We have revised our manuscript to clarify this point: “*The tested alternative alleles and corresponding genotypes in survival analysis were the same as the alternative alleles used in eQTL analysis*.”

During the analysis, we used identical reference/alternative allele definitions from the original VCF and employed the same genotype matrix in both analyses. Each variant is labeled in the format chr_pos_ref_alt (e.g., chr9_42852947_G_A) as a precise variant_id, where G is the reference allele and A is the alternative allele.

In the FastQTL pipeline, the slope is relative to the alternative allele by default (in this example, it refers to“A”). When performing Cox regression for survival analysis, we extracted the same genotype calls from the same original muti-sample VCF file using identical 0/1/2 coding (0=reference homozygote, 1=heterozygote, 2=alternative homozygote). Thus, for both eQTL and survival analyses, the genotype and its corresponding effect are consistently assigned concerning the same alternative allele (“A” in this example).

Consequently, the direction of effect (i.e., slope in eQTL and hazard ratio in survival) is directly comparable across these two analyses, because they refer to the same allele (the alternative allele) in the same set of samples. This ensures that our filtering based on the consistency of direction between eQTL and survival results is valid.

4. Line 343: “Among the 518 eGenes”. Shouldn’t it be 805 eGenes?

*Thank you for identifying this numerical error. You are correct - it should be "805 eGenes" rather than "518 eGenes."*

*We have revised this text to accurately reflect our findings:* “*Among the 805 eGenes in the JP cohort, 321 eGenes are shared in both the JP cohort and the Pan-cancer study (39.9%, p=4.19037e-4, hypergeometric test)."*

*The 518 number referenced in our original text represents JP cohort-specific eGenes (805 total eGenes minus 287 that overlapped with GTEx kidney data), but our intention here was to discuss the proportion of JP cohort eGenes that overlapped with the Pan-cancer study, which is correctly based on the total 805 eGenes.*

5. Line 352-354: The authors cannot attribute 50% of eQTL identified in this dataset as potential “ethnic specific” eQTLs without proper assessment of allele frequencies, for example. There could be multiple other reasons, including differences in the genotype and expression detection methods, imputation methods, tumor histology, grade, somatic drivers, eQTL analysis methods, to name a few.

Thank you for raising this important points. We agree that multiple factors besides ethnicity could contribute to the divergence in eQTL signals detected between our cohort and other studies.

We have revised the text to emphasize that these eQTLs are “unique to our dataset” rather than conclusively “ethnic-specific”: “*There are also more than 50% of eQTL identified in the JP cohort that is not reported in the Pan-cancer study that being the unique finding for this study.”* We also discussed additional plausible reasons for the differences in the discussion section, which reflecting Reviewer #3 ’s suggestions as well: “*These unique eQTL signals from JP cohort should be interpreted carefully due to technical difference among cohorts’ sequencing and analysis methods. On another aspects,…”*

**Reviewer #3**: The manuscript by Song et al reports a moderate yet comprehensive genomic-transcriptomic analysis of 100 Japanese clear cell renal cell carcinoma patients. The authors perform germline eQTL discovery, somatic mutation calling as well as a novel and interesting association analysis with survival. Albeit the moderate size of the cohort, the resource can be extremely valuable to study the differences in genetics ccRCC in different populations. I have the following comments:

• Ref 6, Please also include the most recent larger GWAS of ccRCC (PMID: 38671320).

Thank you for bringing this important reference to our attention. We have included the most recent larger GWAS of ccRCC (PMID: 38671320) as our reference [7].

• Fig 2B: Given the overall lower sample size of the JP cohort, some of the top results might be due to few samples in one genotype group. E.g. For RPS26 CC has two samples(?). So I would encourage the authors to do a further sensitivity analyses for the top findings using dominant/recessive models for each allele to weed out any possibility of low-sample bias results.

Thank you for raising this important concern about potential bias due to small sample sizes in the JP cohort, particularly for variants with unbalanced genotype distributions like RPS26 (CC=2 samples, CG=36 samples, GG=62 samples).

Following your suggestion, we conducted a further sensitivity analyses on our top findings (805 eGenes, with their top eQTLs per gene) under different inheritance models: 1) Additive model: Each minor allele contributes incrementally (0=ref/ref, 1=ref/alt, 2=alt/alt); 2) Dominant model: At least one variant allele shows effect (0=ref/ref, 1=ref/alt or alt/alt)and 3) Recessive model: Two variant alleles required for effect (0=ref/ref or ref/alt, 1=alt/alt). If the minimal sample count among any of the model groups is less than three the pvalue will be shown as NA in the corresponding plot. This methods have been added to our revised manuscript under section 2.5:“*To further assess the robustness of top associations and minimize potential bias due to low sample counts, we performed additional sensitivity analyses using addictive, dominant and recessive genetic models. For each model, we performed simple linear regression (lm(expression ~ genotype)) to evaluate association significance and consistency across models. Top findings that failed to reach significance in all tested models and had fewer than three samples in both the heterozygous and homozygous alternative genotype groups were excluded from further analysis.”*

We further revised results section and Figure legend of **Fig 2B** accordingly.

Regarding RPS26, it is significant under Recessive model so we still kept it in the results. In our revised Fig 2B, we used plots with three different genetic models to substitute the previous plots. We tagged the pvalue from the simple linear models, and also the original qvalue from FastQTL for our top-4-findings.

**Old** Fig 2B:

New **Fig 2B**:

We have revised our corresponding resutls section as well based on this sensitivity analysis that weed out 46 eGenes that may be false positive: “*The top-4 most significant eGenes, LINC01291, ERAP2, XRRA1 and RPS26 with their top-eQTLs were shown in* ***Fig 2B*** *, where further linear regression analysis were performed using addictive, dominant and recessive models to confirm their significant differential expressions…”*

Notably, these 46 genes did not affect our downstream results. They were not included in any of our key gene lists, including the eGene-eSNP pairs tested for directional consistency of effect sizes (54 genes **in Supplementary Table 7**) and the final cross-cohort validated eQTLs (**Supplementary Table 8**). Overall, our downstream filtering and validation steps effectively excluded these potential negative QTL signals, ensuring that they did not influence the conclusions of our study.

• Line 325: Please quantify/mention the “small proportion” of eQTL with more than one gene. Please use quantifications whenever possible rather than using qualitative descriptors.

Thank you for pointing out this. We have revised our texts to “*Among the 4558 unique eQTLs, 90% of them only targets to one eGene and 10 % of eQTLs target more than one eGenes (****Supplementary Fig 4****).”*

• Fig 3. I would suggest adding a plot describing the relation between effect allele frequency and effect size which can inform the extent of selection.

We strongly appreciate your suggestion, and we think that it can be a strong benefit of showing the natural selection, which is quite interesting. We added a series of sub-Figs which compose our new **Fig 3C**, representing the relationship between MAF of our effect allele and the effect size (the absolute value of beta coefficients for each allele). We plotted both all significant eQTLs and the top eQTL each gene, similar to how we did in plotting distributions of distances. We also calculated the Spearman correlation of both, showing a significant negative correlation between MAF and Absolute effect size (Top eQTLs showing bigger negative correlation). In our data, since the relatively small sample size, SNPs that only have a minor allele count of 2 that divided by valid alleles recognized by fastqtl, lead to an MAF close to 0.01, which is plotted on the left side of the plot, with a high effect size. We think it might be some low-frequencies variant that cause significant expression changes.

We have updated the whole new **Fig 3** with legends and also in our manuscript,results section 3.1: “***Fig 3C*** *illustrated the negative association between effect allele frequency and absolute eQTL effect size (beta-coefficients). Spearman correlation analysis revealed a significant negative correlation for both all significant eQTL pairs (ρ = -0.451, p < 1.5e-260) and top eQTL per gene (ρ = -0.599, p < 2.1e-79).* ”

(For visualization, we used: Scatter plots with LOESS smoothing curves to show the overall relationship and Binned analysis where we grouped MAF into ranges (0-0.05, 0.05-0.1, etc.) and plotted the median effect size for each bin)

In Fig 3C the coloring of the density plots overlap which makes the “all eQTL” plot difficult to see.

We have updated the plot (now shown as **Fig 3D**) using a faceted layout with a shared y-axis to improve comparison. The results remain consistent: top eQTLs are enriched in regions closer to the TSS compared to all eQTLs.

Fig 3D: “Lorem ipsum”?

Thank you for catching this oversight. We have removed the "Lorem ipsum" text from new version of **Fig 3E** in the revised manuscript.

• For Fig 3B-C, given that this is a WES study, how is the major portion of variants intron variants and substantial effects exist in the regulatory regions?

Thank you for raising this concern. The key reason of this is that we utilized both on-target and off-target captures from the WES data to get more high-quality regulatory variants calls. Specifically, we included variants with <25% missingness that were flanking intronic regions near designed exons. Also, we used a cis-window of ±1 Mb around the transcription start site (TSS), which extends well beyond the captured exonic sequences, thereby naturally covers many variants from intron and non-coding regions. The variants inside this window contains significant proportions (83.7%) of non-coding region variants (**Supplementary Fig 2**). The regulatory signals we identified in non-coding regions were also consistent with recent findings in other datasets, indicating that many regulatory variants reside in non-coding regions.

Following by the discussions with Reviewer #1 and #2, our design of WES is based on majorly three previous published studies: Guo et al. (6) demonstrated that “a significant amount of high quality genotypes outside target regions can be obtained from exome sequencing data “, especially for Agilent SureSelect kit , they ” obtained 84,049 high-quality SNPs outside target regions compared to 65,231 SNPs inside target regions (a 129% increase).”Díaz-de Usera et al., (7) confirmed that “Oﬀ-target reads, which could represent 40–60% of the reads in a WES study, can also be a source of informative genetic variation” by utilizing the GATK variant discovery pipeline, the same as employed in our study. Meienberg et al. (8)evaluated off-target regions which defined as more than 500 bp outside the designed target regions, showed that WES effectively captures variants in UTRs, promoter-proximal regions, and exon-flanking intronic sequences.

Overall, the combination of the fact that off-target capture features of WES platforms and the broad cis-window used for eQTL mapping explains why a major portion of the variants—and their associated regulatory effects—are observed in intronic regions even in a WES-based study. We hope this explanation clarifies the rationale behind our previous findings in old Fig 3B-C.

• Line 349: Is there a typo in the p-value? In general, I am not sure about how precise the hypergeometric test is when testing for overlap of significant eQTLs which are strongly correlated.

The p-value originally stated in the manuscript is not a typo but means an extremely small p-value (showing value 0) that, in RStudio, if the p-value is lower than 1.11𝑒−16. As the reviewer suggested, we removed the results of the hypergeometric test for SNP data, because of sequencing coverage differences and the Linkage Disequilibrium (LD) phenomenon. This might be the cause of we got extremely small p-value, and also be an obstacle to performing statistical tests among these SNP sets. Instead, we only listed the overlaps that JP eqtl we identified that are also shown in the GTEx/Pancancer study (**Supplementary Fig 5A-B**), to clearly show how many eqtls were identified previously (on healthy kidney and TCGA-KIRC) and how many were newly identified.

This is our intrinsic intention in comparing these SNPs among different datasets. Regardless, the eGene results are not affected by sequencing coverage or approaches and show highly significant overlaps between JP eGenes and those identified in GTEx and Pan-cancer databases on Fig 3E.

• Line 352: The population specificity might be due to a plethora of reasons: (a) the variant is absent in one population (b) the effect allele frequencies differ (c) the LD structures differ (d) the eQTLs indeed have different effects. To me the most interesting scenarios are (a) and (d). Can the authors comment on these?

Thank you for this insightful comment. We agree that the scenario (a) and (d) would be most interesting. Our study revealed several interesting patterns:

(1) Even with GTEx's comprehensive WGS coverage, we identified unique eQTLs not detected in GTEx healthy kidney tissues, this might be reflecting more on cancer-specific regulation, reflecting scenario d. But that could also be because of scenario (a), that these SNPs were absent from GTEx kidney cohort (WGS but lower than 100 samples).

(2) JP-specific variants absent in TCGA may be due to genetic differences between populations or statistical power limitations due to some alleles being relatively low, so limited the statistical significance testing, which reflects more for scenarios (a) and (b).

(3) Our analysis confirmed that the variants with lower minor allele frequencies show substantially larger effect sizes, though their rarity limits detection in conventional analyses, reflecting scenario (a). Since these variants seem to have big eQTL signals, we think big-cohrt and deep sequencing analysis on future studies will be crucial.

(4) Significant overlap in eGenes across all cohorts suggests conserved regulatory mechanisms among populations, which seems to be a housekeeping regulatory mechanism.

We acknowledge that scenario (c) regarding LD structure differences warrants detailed investigation beyond our current scope. Future trans-ethnic fine-mapping studies will be essential to fully resolve these mechanisms. We have incorporated these insights into **the discussion section**.

• The discussion on section 3.3 should be represented by a somatic mutation Fig.

Thank you for the suggestion. We have added a new **Fig 4A** to illustrate somatic mutation findings in section 3.3. Upon re-examination, we identified 712 (not 748) recurrently mutated genes (revised **Supplementary Table 4**) and 19 (not 13) somatically mutated eGenes (**Fig 4B**). We also showed detailly these 19 genes in **Fig 4C**, with their corresponding qvalue from eQTL analysis. We have revised accordingly*:*“*A total of 19 significant eGenes of JP cohort overlapped with highly recurrent somatic mutated genes (at least 5 times), as shown in* ***Fig 4C****. Among them, DNAH11 showed the highest somatic mutation load (19 mutations) and the most significant eQTL signals (by q-value).*”

• Line 422: Typo: “derived”?

Thank you for pointing out this typo error. Instead of “deprived” it should be “derived”. We have corrected it in the revised manuscript to *“To further explore the consistency of prognostic values of eGene-eQTL pairs, we performed a parallel survival analysis integrating the slope of allelic effect* ***derived from*** *eQTLs association analysis.”*

• I would commend the authors for nicely hypothesizing and summarizing the integration of eQTLs in survival analysis.

Thank you for your positive feedback. We appreciate your commendation and are glad you find our hypothesis and approach to integrating eGenes-eQTLs into the survival analysis valuable. This integration represents a major novelty of our study.

• Although the replication of survival association in TCGA is limited, I would recommend the authors perform a slightly lenient search in terms of similar direction of effects.

Thank you for this thoughtful suggestion regarding our replication approach. We agree that adopting a more lenient criterion for replication would be valuable given the limited overlap in significant survival associations between cohorts.

As shown in our new direction of effects comparison Fig (**Fig 6A**), we have implemented both strict and lenient replication categories. The strict replication (green points) requires both statistical significance and consistent effect direction across cohorts, while the lenient replication (orange points) focuses primarily on consistent effect direction regardless of significance threshold in TCGA.

This approach has allowed us to identify additional variants with consistent directional effects that might not reach statistical significance in TCGA due to various factors including cohort size, genetic background differences, or treatment variations. The Fig clearly demonstrates several variants in the upper-right and lower-left quadrants showing directional consistency (falling near the diagonal line), supporting your recommendation for this more inclusive approach to cross-population validation.

We revised our methods (section 2.8) and results section(section 3.5) to reflect this analysis, accordingly.

I would also strongly recommend the authors compare the results with the TWAS reported in PMID: 39137781, which can give a complementary evidence.

Thank you for suggesting this comparison with the TWAS reported in PMID: 39137781. We have conducted this analysis and found valuable complementary evidence that strengthens our findings. We presented the results on **Supplementary Fig 10 as well as results section 3.6**.

As illustrated in Supplementary Fig 10A, we identified 15 overlapping genes between our JP eGenes and the TWAS genes from this study (11 general overlap plus 4 that are also prognostic). This overlap, while modest in absolute numbers, represents significant biological convergence across different analytical approaches and populations. Supplementary Fig 10B further details the distribution of these 15 overlapping genes across various tables in our analysis. Supplementary Fig 10C specifically highlights the 4 genes that overlap between TWAS genes and our potential prognostic eGenes list.

This comparison provides some extra interesting genes pops out from this study.

• Fig 6. Direct screenshots from GTEx server is not recommended to be included as a main Fig. This can go in as text and/or supplementary as it is not the work of the authors directly.

Thank you for this comment. Now we substituted those Figs into the text descriptons in the manuscript, as “… *the AA genotype is associated with lower expression of* ***ANKRD20A7P*** *in the JP cohort and GTEx database in kidney tissues with the same allelic effect directions (****Fig 7A,Supplementary Fig 9A****),*” as well as a **Supplementary Fig 9:**. See belows as our new **Fig 7**:

**Reference**

1.	Moiso E. Manual curation of TCGA treatment data and identification of potential markers of therapy response [Internet]. medRxiv; 2021 [cited 2025 Apr 23]. p. 2021.04.30.21251941. Available from: https://www.medrxiv.org/content/10.1101/2021.04.30.21251941v2

2.	Uhlen M, Zhang C, Lee S, Sjöstedt E, Fagerberg L, Bidkhori G, et al. A pathology atlas of the human cancer transcriptome. Science. 2017 Aug 18;357(6352):eaan2507.

3.	Nagy Á, Munkácsy G, Győrffy B. Pancancer survival analysis of cancer hallmark genes. Sci Rep. 2021 Mar 15;11(1):6047.

4.	Liu X, Liu P, Chernock RD, Kuhs KAL, Lewis JS, Luo J, et al. A prognostic gene expression signature for oropharyngeal squamous cell carcinoma. eBioMedicine [Internet]. 2020 Nov 1 [cited 2025 Apr 24];61. Available from: https://www.thelancet.com/journals/ebiom/article/PIIS2352-3964(20)30180-8/fulltext

5.	Smith JC, Sheltzer JM. Genome-wide identification and analysis of prognostic features in human cancers. Cell Reports [Internet]. 2022 Mar 29 [cited 2025 Apr 24];38(13). Available from: https://www.cell.com/cell-reports/abstract/S2211-1247(22)00313-8

6.	Guo Y, Long J, He J, Li CI, Cai Q, Shu XO, et al. Exome sequencing generates high quality data in non-target regions. BMC Genomics. 2012 May 20;13(1):194.

7.	Díaz-de Usera A, Lorenzo-Salazar JM, Rubio-Rodríguez LA, Muñoz-Barrera A, Guillen-Guio B, Marcelino-Rodríguez I, et al. Evaluation of Whole-Exome Enrichment Solutions: Lessons from the High-End of the Short-Read Sequencing Scale. J Clin Med. 2020 Nov 13;9(11):3656.

8.	Meienberg J, Zerjavic K, Keller I, Okoniewski M, Patrignani A, Ludin K, et al. New insights into the performance of human whole-exome capture platforms. Nucleic Acids Research. 2015 Jun 23;43(11):e76.

9.	Hoffman JD, Graff RE, Emami NC, Tai CG, Passarelli MN, Hu D, et al. Cis-eQTL-based trans-ethnic meta-analysis reveals novel genes associated with breast cancer risk. PLOS Genetics. 2017 Mar 31;13(3):e1006690.

10.	Ni J, Deng B, Zhu M, Wang Y, Yan C, Wang T, et al. Integration of GWAS and eQTL Analysis to Identify Risk Loci and Susceptibility Genes for Gastric Cancer. Front Genet [Internet]. 2020 Jul 10 [cited 2025 Apr 27];11. Available from: https://www.frontiersin.orghttps://www.frontiersin.org/journals/genetics/articles/10.3389/fgene.2020.00679/full

11.	Xu H, Wu Y, Chen Q, Yu Y, Meng Q, Qin N, et al. Integrating apaQTL and eQTL analysis identifies a potential causal variant associated with lung adenocarcinoma risk in the Chinese population. Commun Biol. 2024 Jul 13;7(1):1–13.
