# Supplementary material for: Systematically identification of survival-associated eQTLs in a Japanese kidney cancer cohort
Source: PLoS Genet. 2025 Jul 7;21(7):e1011770. doi: 10.1371/journal.pgen.1011770 (PMC12233309; doi:10.1371/journal.pgen.1011770)
Supplement: Attachment — Submitted filename: Response to Reviewers_3rd_PGENETICS-D-25-00018R2.docx [file pgen.1011770.s023.docx]

Dear editors,

We would like to thank you and the reviewers for the thorough evaluation of our manuscript (Manuscript Number: PGENETICS-D-25-00018R2) titled "Systematically identification of survival-associated eQTLs in a Japanese kidney cancer cohort".

During the last round of revision, we already adjusted the manuscript text to address the suggestions from **Reviewer #1,** and we believe that we have adequately addressed the concern. For your convenience, we have added the line number of the place where the text is being changed in our tracked change manuscript.

We did not receive any further suggestions from the email, so we would like to ask you to review our point-by-point response below from the last submission. If you have any new requirements, please feel free to contact us.

**Below is the content from our last revision(PGENETICS-D-25-00018R2):**

We provided point-by-point responses to the reviewers' comments, mainly from Reviewer #1.

In particular, we reported hazard ratios (HRs) and confidence intervals for top eQTLs in a new Supplementary Table 6. Additionally, we expanded our discussion section based on suggestions and referred to more previously published studies to strengthen the scientific contributions of our manuscript.

We also corrected a minor numerical error in the abstract: the number of significant prognostic eGenes is 9 (not 10), accounting for one overlapped gene between the unadjusted (n=7) and adjusted (n=3) models.

All corresponding revisions to the main text, figures, and supplementary materials are included in the tracked version of the manuscript.

We believe these revisions have greatly improved the manuscript, making it more focused and scientifically robust. We hope that the revised version meets the expectations of both the editors and the reviewers. We look forward to hearing from you and are prepared to make any further revisions as needed.

Thank you once again for the opportunity to revise and improve our work.

Sincerely,

Adil Mardinoglu	Cheng Zhang

Professor of Systems Biology	Lecturer, Researcher

[adilm@scilifelab.se](mailto:adilm@scilifelab.se)	[cheng.zhang@scilifelab.se](mailto:cheng.zhang@scilifelab.se)

adil.mardinoglu@kcl.ac.uk

SciLifeLab, Science for Life Laboratory,	SciLifeLab

KTH-Royal Institute of Technology,	KTH

Stockholm, Sweden	Stockholm, Sweden

Centre for Host-Microbiome Interactions	The Roger Williams Institute of Liver	Studies

Faculty of Dentistry, Oral & Craniofacial Sciences	Faculty of Life Sciences & Medicine

King's College London, UK	King's College London, UK

http://sysmedicine.com

**Point-to-point response to reviewers’ comments:**

We thank all reviewers for their valuable comments and constructive suggestions. Below, we provide point-to-point responses to each comment in blue font, with corresponding manuscript text shown in green font. All changes in the main text are tracked.

Reviewer #1: This study systematically identifies survival-associated expression quantitative trait loci (eQTLs) in a Japanese clear cell renal cell carcinoma (ccRCC) cohort and validates findings in the TCGA-KIRC dataset. The work is well-designed, methodologically sound, and provides novel insights into the prognostic role of germline regulatory variants in ccRCC, particularly in an understudied Asian population. However, several issues require clarification and improvement to strengthen the manuscript’s impact and reliability.

1. Caution is needed when attributing unique eQTLs to ancestry (e.g., "50% are Asian-specific"). Differences could stem from technical factors (e.g., WES coverage) or tumor-specific regulation.

Thank you for raising this concern. We fully agree with this point.

**[Line 101-102]** We revised the “Asian-specific” texts in our introduction to:” We first compared our results with existing kidney and ccRCC-specific databases, including GTEx release version 8 (v8) and a Pan-cancer eQTL database, to identify the shared ccRCC eGenes/eQTLs across all three cohorts and the eQTLs unique to the JP cohort”.

**[Line 411-412]** In the first round of revision, we have revised the “50% are Asian-specific” texts in our results section to: “There are also more than 50% of eQTL identified in the JP cohort that is not reported in the Pan-cancer study that being the unique finding for this study.”

**[Line 1052]** Also in the conclusion section, we deleted the sentence “We identified both ethnicity-specific and cancer-specific eQTLs.”

**[Line 615-621]** Lastly, we have discussed additional plausible reasons for the differences in the discussion section, which also reflects previous Reviewer #3 ’s suggestions (In the first round of revision). We also reordered the sentences for greater clarity: “These unique eQTL signals from the JP cohort should be interpreted carefully due to technical differences among the cohorts’ sequencing and analysis methods. On another aspect, these differences could also … underscoring the need for larger and deeper sequencing studies.”

We cautiously checked that there are no sentences including judgment of “Asian-specific” in our revised manuscript.

2. Report hazard ratios (HRs) and confidence intervals for top eQTLs to assess clinical relevance.

**[Line 483-491]** Thank you for this valuable suggestion. We have reported Cox regression analysis results (unadjusted and adjusted models) for all top eQTLs corresponding to our identified eGenes (n=805) in a new **Supplementary Table 6.** The methods of doing Cox regression analysis remain the same as we did before. We added it to our results section 3.4: “Firstly, the hazard ratios (HR) and 95% confidence intervals (CI) for all of the top-eQTLs were reported in **Supplementary Table 6.** …”

Since there is indeed a top-4 eQTL with a P<0.05 signal (rs3771800(T>C)-LINC01291), we mentioned it in the results section: “Notably, rs3771800(T>C)-LINC01291, as one of …” and the corresponding discussion: “In our findings, rs3771800 CC increases the LINC01291 expression, however, showing protective signals to ccRCC, which suggests…”

All other analysis remain unchanged.

3. Fig 6A: Label axes with effect directions (e.g., "Protective" vs. "Risk" alleles).

**[Fig 6A]** Following your suggestion, we added arrows and explanation texts for the x-axis and y-axis to clearly label with effect directions.

New **Fig 6A**: showing the labels by light red as risk direction and light blue as protective direction.

4. Ensure all cited tables/figures (e.g., somatic mutations in Suppl. Table 4) are referenced in the main text.

We checked and confirmed that all cited tables/figures are referenced in the main text.

Suppl. Table 4 was referred to in the results section 3.3: “A total of 712 mutated genes with at least five recurrent mutations have been identified (**Supplementary Table 4**).”

5. In discussion: Compare findings to recent ccRCC GWAS (e.g., PMID: 38671320) and TWAS (e.g., PMID: 39137781).

The TWAS study (PMID: 39137781) was compared in the result section **Supplementary**

**Fig 10,** as well as the results section 3.6.

We now compared our eQTL results with recent ccRCC GWAS of ccRCC (PMID:

38671320), mainly their Supplementary Table 3 with 108 loci independently associated with kidney cancer.

Interestingly, rs12513763 is both a GWAS locus from the literature and an eQTL from our study (regulating SLC6A18), which represents this SNP both case-control significance and gene regulation significance. On the gene level, NTN4 is shown in both their Supplementary Table 3 results and our eQTL analysis.

**[Line 770-779]**We have added a section on discussion to mention these interesting overlaps and refer to some of the previous studies that mentioned the corresponding genes: “By comparing with a recent published kidney-cancer GWAS[6] and TWAS[36] study, several overlapped signals were emphasized. Firstly,rs12513763 emerged…”

6. Discuss why chromosome 9p11.1 emerges as an eQTL hotspot (e.g., open chromatin, high gene density).

**[Line 1003-1009]** Thank you for this valuable suggestion.

In our discussion, we have further investigated the potential biological mechanisms underlying the 9p11.1 as an eQTL hotspot: “Centromeres are often enriched for non-coding RNAs, pseudogenes, and repetitive elements … such as during cancer cell development”. Drawing from previous studies, we suggest that the centromeric region’s high gene density per cM, abundance of pseudogenes and ncRNAs, and potential open chromatin configuration may contribute to its potential regulatory effects. Interestingly, we also found a newly published paper reported trans-eqtl hotspots on the chr1 centromere region, so we added it to our discussion. Relevant citations have been added to support this explanation.

The manuscript is well-written and analytically thorough but requires clarifications on statistical methods, biological context, and presentation. No additional data is needed.

As you see in our tracked manuscript, we also revised some minor grammatical words, sentences that may be unclear, to enhance our methods and discussions sections. We hope that our revision helped to enhance these aspects.

Reviewer #2: The authors addressed most of the points raised by this referee.
Minor comments:
Supplementary Fig2A: please specify what the "regulatory" category includes in the legend.

We specified this in our supplementary Fig. 2A legends:” **Supplementary Fig 2. Genomic annotation and distribution of tested variants in cis-eQTL analysis.** (A) Proportions of variants in intronic (60.9%), coding (16.3%), …The "intronic" category includes intron variants, while "intergenic" refers to variants annotated as intergenic. Variants not fitting these classifications were grouped as "other non-coding".

Table 2: I suggest that the authors use the term "significant" instead of "solid" to describe their findings.

Thank you for this suggestion. We modified our texts and table title accordingly:” there are 7 significant prognostic eGenes identified in the univariate Cox analysis and 3 prognostic eGenes in the adjusted Cox analysis (Table 2), which sum to a total of 9 unique eGenes”.

Reviewer #3: The authors have addressed all my queries satisfactorily. I congratulate them on this important work.

Thank you so much.
